# Chemoattraction of bone marrow-derived stem cells towards human endometrial stromal cells is mediated by estradiol regulated CXCL12 and CXCR4 expression

**DOI:** 10.1016/j.scr.2015.04.004

**Published:** 2015-04-24

**Authors:** Xiuli Wang, Ramanaiah Mamillapalli, Levent Mutlu, Hongling Du, Hugh S. Taylor

**Affiliations:** Department of Obstetrics, Gynecology and Reproductive Sciences, Yale School of Medicine, New Haven, CT, USA

## Abstract

Bone marrow derived cells engraft to the uterine endometrium and contribute to endometriosis. The mechanism by which these cells are mobilized and directed to the endometrium has not been previously characterized. We demonstrate that human endometrial stromal cells (hESCs) produce the chemokine CXCL12 and that bone marrow cells (BMCs) express the CXCL12 receptor, CXCR4. Treatment with physiological levels of estradiol (E2) induced both CXCL12 and CXCR4 expression in hESCs and BMCs, respectively. BMCs migrated towards hESCs conditioned media; a CXCR4 antagonist blocked migration indicating that CXCL12 acting through its receptor, CXCR4, is necessary for chemoattraction of BM cells to human endometrial cells. E2 increased both CXCL12 expression in endometrial cells and CXCR4 expression in BM cells, further enhancing chemoattraction. E2 induced CXCL12/CXCR4 expression in endometrium and BM, respectively, drives migration of stem cells to the endometrium. The E2-CXCL12/CXCR4 signaling pathway may be useful in determining treatments for endometrial disorders, and may be antagonized to block stem cell migration to endometriosis.

## Introduction

CXCR4 belongs to the CXC family of chemokine receptors. Interaction of CXCR4 with its ligand, stromal derived factor (SDF-1α, CXCL12) plays a key role in the mobilization and homing of stem cells (Hopman and DiPersio, 2014). CXCR4, expressed on the surface of stem cells, serves as a target for modulating migration (Lai et al., 2014). CXCL12 is produced by the stromal cells and endothelial cells of many organs including bone marrow (BM), endometrium, skeletal muscle, liver and brain (Sharma et al., 2011). In human endometrium, CXCL12 is expressed by stromal cells. Estradiol (E2) stimulates CXCL12 production from endometrial stromal cells (ESCs) (Ruiz et al., 2010; Tsutsumi et al., 2011) suggesting a role in stem cell recruitment to the uterus.

BM-derived cells including hematopoietic stem cells (HSCs), mesenchymal stromal cells (MSCs), and endothelial progenitor cells (EPCs), significantly contribute to peripheral tissue repair and angiogenesis (Beauséjour, 2007). Therefore, factors influencing BM-derived cell migration and function are likely to have a broad impact. Overexpression of CXCR4 in stem cells (by cytokine induction or gene transfection) enhances MSCs homing *in vivo* to bone marrow as well as migration *in vitro* towards CXCL12 (Shi et al., 2007; Liu et al., 2013a; Marquez-Curtis et al., 2013; Hu et al., 2013). Recently it has been demonstrated that estrogen receptor (ER) is expressed in EPCs *in vivo* and *in vitro* (Baruscotti et al., 2010). EPCs proliferation is induced during the menstrual phase and the proliferation can be affected by estrogen through ERα activation (Foresta et al., 2010). These studies suggested the potential regulation of stem cells by sex steroids. Previous studies from our laboratory showed that BM-derived stem cells can engraft in the murine endometrium (Du and Taylor, 2007). We have shown that ischemia–reperfusion injury, toxicant exposure, and medications can alter the migration of BM-derived stem cells to the uterus, however the molecular mechanism responsible for the recruitment and engraftment of these cells is unknown (Zhou et al., 2011; Sakr et al., 2014; Lapidot, 2001). Here we report the effects of female sex hormones estradiol and progesterone on CXCR4 and CXCL12 expression, and the role of this chemokine and its receptor in migration of BMCs towards hESCs.

## Material and methods

### Cell culture

Mouse bone marrow cells (mBMCs) were prepared from 8–10 weeks old female C57 BL/6 mice (Charles River Laboratories, Wilmington, USA) by flushing bone marrow from the tibia and femur, and filtering the marrow through sterile 70-μm nylon mesh. The filtered mBMCs were grown at a density of 2.5 × 10^6^ cells/ml in DMEM/F-12 medium supplemented with 15% fetal bovine serum, containing penicillin (100 μg/ml) and streptomycin (100 μg/ml) (GIBCO-BRL, Rock-ville, USA). After 48 h the cells were gently washed with PBS and fresh medium added; the medium was subsequently changed for every 3–4 days until two weeks when the cells were used for experiments described below. Mouse uterine cells (mUCs) were prepared from 6–8 weeks old female C57 BL/6 mice by enzymatic digestion of the uterus in 0.125% type IA collagenase (Sigma, USA) for 1 h at 37 °C, and then filtered through a 70-μm filter. Human endometrial stromal cells (hESCs) were obtained from human endometria in the proliferative phase as described by Ryan et al. (1994). Both mUCs and hESCs were cultured in DMEM/F12 medium supplemented with 10% FBS and penicillin/streptomycin (100 μg/ml) for one week. The cells were then washed with PBS, trypsinized, plated and cultured for an additional 48 h before carry out the experiments. Experiments used to obtain the mouse and human cells were conducted under approved Yale Institutional Animal Care and Use Committee and Human Investigations Committee protocols, respectively.

### ABC-immunocytochemistry (ICC) and fluorescent ICC

Cells grown (80% confluent) on glass microscope slides were fixed with freshly prepared 4% formaldehyde for 10 min and rinsed three times for 5 min each with PBS. The cells were blocked with 4% BSA in PBS for 30 min and incubated with the primary antibody (diluted in 1% BSA in PBS) in a humidified chamber overnight at 4 °C. For ABC-ICC, the cells were incubated with the secondary antibody in 1% BSA for 30 min at room temperature. The ABC staining and 3, 3′diaminobenzidine (DAB) kits (Vector Laboratories, USA) were used to visualize the immunocytochemical reaction under light microscope (Olympus BX41). For fluorescence-ICC, the cells were incubated with the secondary antibody in the dark for 30 min at room temperature and 4′, 6-diamidino-2-phenylindole (DAPI) (Vector laboratories, UK) was added on to the cells. The slides were examined under inverted fluorescence microscope (Axiovert 200 M, Zeiss Company, Germany).

### Flow cytometry

After two weeks of culture, mBMCs were analyzed for mesenchymal stromal cells (MSCs), and endothelial progenitor cells (EPCs) by flow cytometry. The cells were incubated with the fluorescent-labeled antibodies against CD90, CD105, CD34, Flk-1 (BioLegend, San Diego, USA) and CD31 (eBiosciences, USA), or with isotype-matched irrelevant antibody (1 μg for 10^6^ cells) for 30 min on ice in dark. The cells were then washed with flow cytometry staining buffer 3 times for 5 min at 3,000 rpm and the cell pellet was resuspended in 1 ml ice cold staining buffer for cell sorting. Flow acquisition was performed on LSRII Fortessa, LSRII, or FACSCalibur analyzers (BD Biosciences), and data were analyzed using Diva software (BD Biosciences, USA).

### Detection of CXCL12 by ELISA

CXCL12α was assayed from the supernatants of cell cultures using ELISA kit (R & D Systems, USA) according to the manufacturer's instructions. mBMC, hESCs and mUC were cultured in DMEM/F12 supplemented with 10% FBS and 1% penicillin and streptomycin in a 6-well plate (1 × 10^5^ cells/well). The supernatants were collected from 48 h old cell cultures. For steroid treatment, the 48 h old mBMC and hESCs cells were serum starved overnight and treated for 24 h with E2 or progesterone (P4) (Sigma, USA) at concentrations of 1 × 10^−8^, 1 × 10^−7^, 1 × 10^−6^ M. The supernatants were then collected.

### Migration assay

The migration assay for mBMC and hESC cells was carried out using 8-μm pore size polycarbonate membrane (Millipore, USA). The serum free conditioned medium (600 μl) collected from 48 h old cultures from both cell types was added into the lower chamber and 200 μl of cells (5 × 10^4^ cells) was placed into the upper insert. The cells in the upper insert were serum starved overnight and treated with either E2 for 24 h at 1 × 10^−7^ M, or AMD3100 antagonist of CXCR4 for 30 min before the migration assay. After 16 h in a humidified CO_2_ incubator at 37 °C, the non-migrating cells were scraped with a cotton swab from the top of the membrane. The cells migrating across the membrane were fixed, stained, and counted. Results are reported as chemotactic index (CI), defined as the number of cells migrating in response to the conditioned supernatants divided by number of cells that responded to the serum-free DMEM/F12 medium. Ethanol was used as a vehicle control to exclude the nonspecific effects on CXCR4.

### Detection of CXCR4 by Western blot

Protein extracts (25 to 30 μg) from different cells, as well as treated mBMCs, were subjected to SDS-PAGE and immunoblotting using standard methods. Anti-CXCR4 and anti-α-tubulin antibodies used were from Santa Cruz Biotechnology (Dallas, USA) while secondary antibody conjugated with horseradish peroxidase was obtained from Cell Signaling (Beverly, Massachusetts). The CXCR4 protein band densities were quantified using Quantity One software from BioRad and relative band density was calculated as a ratio of sample to α-tubulin.

### Quantitative real-time RTPCR

RNA was isolated using TRIzol (Invitrogen, Carlsbad, California) and purified on RNeasy minicolumns (QIAGEN, Valencia California), with on-column deoxyribonuclease digestion, as per the manufacturers' instructions. First-strand cDNA was reverse transcribed using iScript cDNA Synthesis Kit while iQ SYBR Green Supermix (Bio-Rad, Hercules, USA) based assays were performed for mCXCR4, mCXCL12 and α-tubulin for qRT-PCR analysis. The CXCR4 primers were as follows: forward 5′-TTTCAGATGCTTGACGTTGG-3′; and reverse 5′-GCGCTCTGCATCAGTGAC-3′; the CXCL12 primers were, forward 5′-ACTCACACTGATCGGTTCCA-3′ and reverse 5′-AGGTGCAGGTAGCAGTGACC-3′ and the primers for α-tubulin were, forward, 5′-ATGGAGGGGAATACAGCCC-3′ and reverse, 5′-TTCTTTGCAGCTCCTTCGTT-3′. For each experimental sample, a control without reverse transcriptase was run to verify that the amplification product arose from cDNA and not from genomic DNA. The relative expression levels normalized to α-tubulin, were determined using the comparative *C_T_* method (also known as the 2^ΔΔ*CT*^ method) (Applied Biosystems, 1997; Barr and Manning, 1999).

### Statistics

Results are presented as the mean ± S.D. Statistical significance was determined using one-way ANOVA with the Newman–Keuls multiple comparisons test. All statistical analyses were carried out using Graph Pad Prism 4.00 for Macintosh (Graph-Pad Software for Science Inc., San Diego, CA, USA).

## Results

### Characterization of mBMC

**Figure 1:**
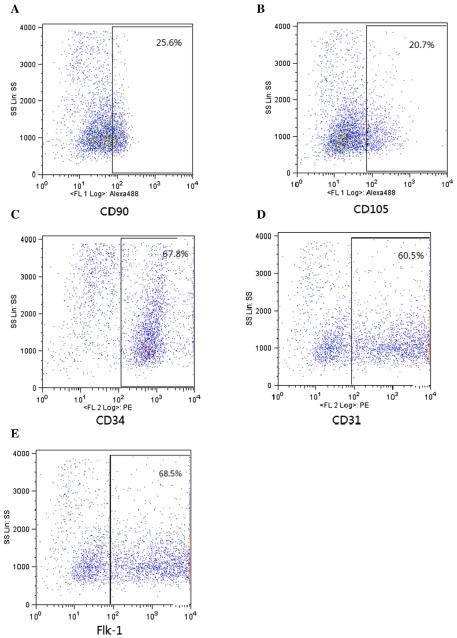
Immunophenotypic characterization of mBMCs by FACS. Primary mBMCs were incubated with fluorescent-labeled CD90, CD105, CD34, CD31, and Flk-1 antibodies or with isotype-matched irrelevant antibody for FACS analysis. Experiments were performed 3 times with different samples and each time in duplicate. Results (% of cells) presented are the average of triplicates.

The CXCR4 and CXCL12 genes are remarkably conserved across diverse species. The human and murine CXCL12 differs by one amino acid and is cross reactive (Lapidot, 2001), providing us with an opportunity to conduct the study of murine CXCR4 with human CXCL12 signaling. The mBMCs were cultured for two weeks, washed with PBS, and trypsinized. The cell pellet was resuspended in FACS staining buffer and incubated with fluorescent labeled antibodies against CD90, CD105, CD34, CD31 and Flk-1. The cells were then and analyzed by FACS. As shown in Fig. 1A, 25.6% of mBMCs expressed CD90 while CD105 (Fig. 1B), CD34 (Fig. 1C), CD31 (Fig. 1D) and Flk-1 (Fig. 1E) were expressed on 20.7%, 67.8%, 60.5% and 68.5% of mBMCs respectively. CD90^+^ and CD105^+^ were considered MSC-specific surface markers while CD34^+^, CD31^+^, and Flk-1^+^ represented the EPC.

### Expression of CXCR4 and CXCL12 in mBMC, mUC and hESCs

**Figure 2:**
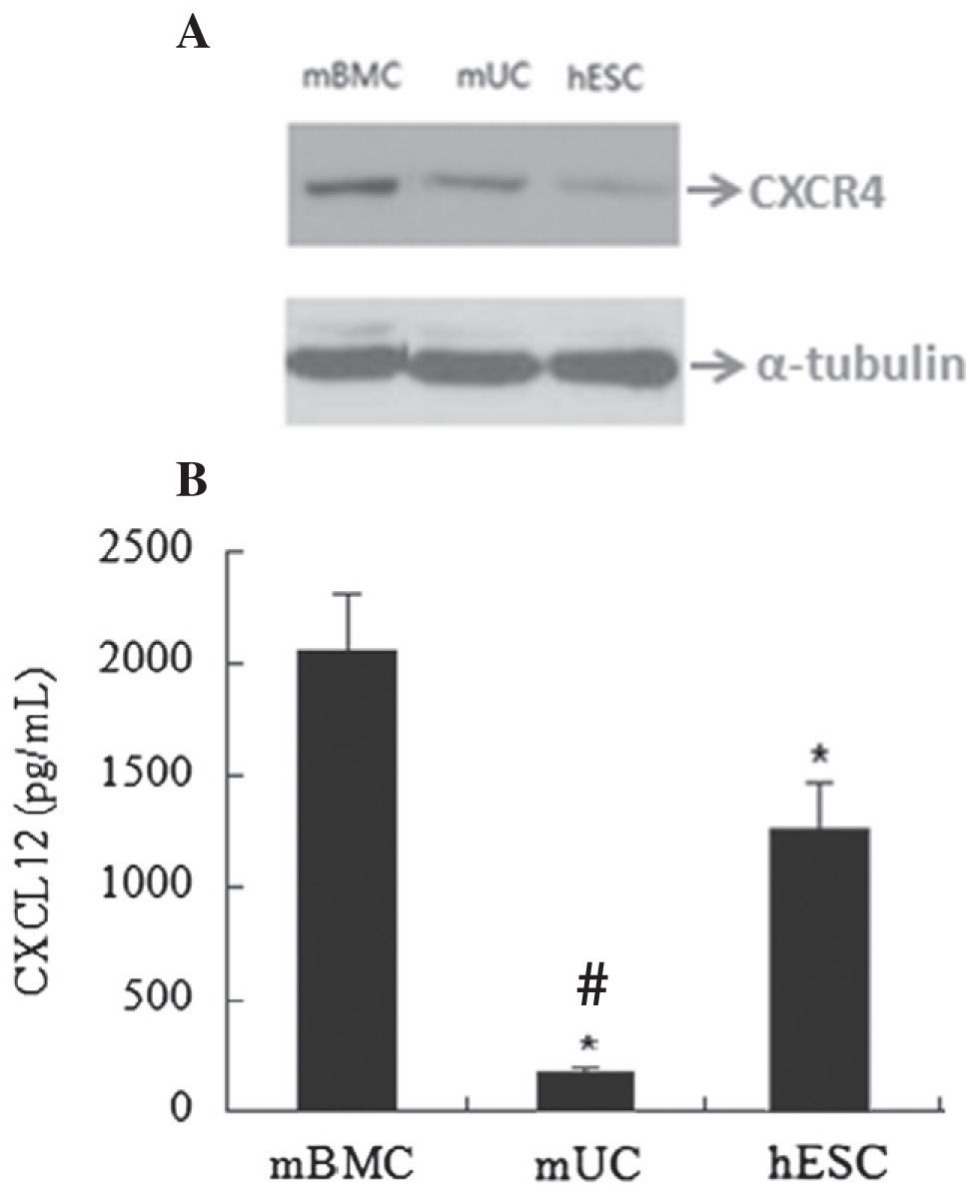
Western blot analysis of CXCR4 and quantification of CXCL12 by ELISA. (A) The CXCR4 protein (25 μg) from mBMC, mUC or hESCs was subjected to 10%-SDS-PAGE and immunoblotted against anti-CXCR4 antibody. (B) The expression levels of CXCL12 quantified by ELISA from the supernatants of 48 h old mBMCs, hESCs and mUCs. The graph presents the mean of three separate experiments; error bars represent the ± S.D. * denotes a statistically significant difference (p b 0.05) vs mBMCs and # denotes a statistically significant difference (p b 0.02) vs hESCs.

Cell lysates were prepared from 48 h old cells and 25 μg of protein from each cell type was subjected to SDS-PAGE followed by immunoblotting. As shown in Fig. 2A mBMCs had the highest CXCR4 expression among the three cell types while lowest expression was seen in hESCs. The differential expression of CXCR4 protein levels among these cell types was correlated with mRNA levels confirmed by qRT-PCR. The density of specific bands was quantified using Quantity One software. The relative band density was calculated as a ratio of sample to α-tubulin (data not shown). The CXCL12α was measured from the conditioned medium collected from the 48 h old cells using ELISA kit. As shown in Fig. 2B, CXCL12 was predominantly expressed in mBMCs; however hESCs also expressed CXCL12 at high level while mUCs showed very low level, CXCL12 expression.

**Figure 3:**
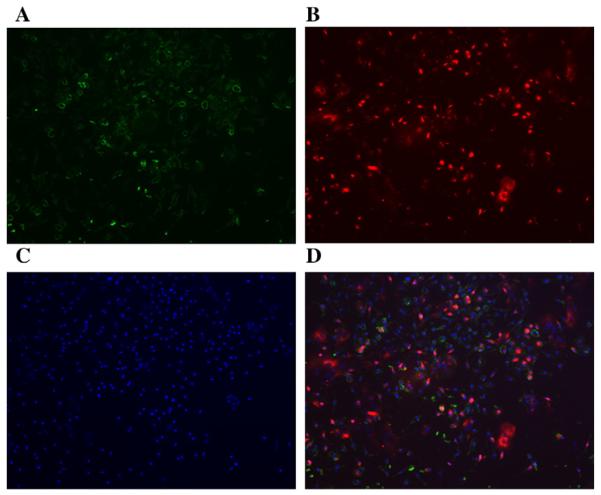
The detection of CXCR4 and CD45 in mBMCs with fluorescent ICC. Representative photomicrographs of fluorescent ICC for CD45 (A, green) and CXCR4 (B, red) expression in mBMCs (100x), DAPI-stained nuclei are depicted in blue (C), and merged CD45 and CXCR4 are shown in (D).

We localized the expression of CXCR4 in mBMCs with fluorescent ICC. As shown in Fig. 3, the CXCR4 is expressed intracellularly in 37.5% of mBMCs. Fig. 3A shows CD45 expression on mBMCs while 3B shows CXCR4 expression; 3C shows DAPI staining for nuclei and 3D shows the merge of CD45 and CXCR4 expression. CXCR4 expression is predominantly seen in the CD45 negative cells.

### Migration of mBMC and hESCs towards chemotactic activity of conditioned medium

**Figure 4:**
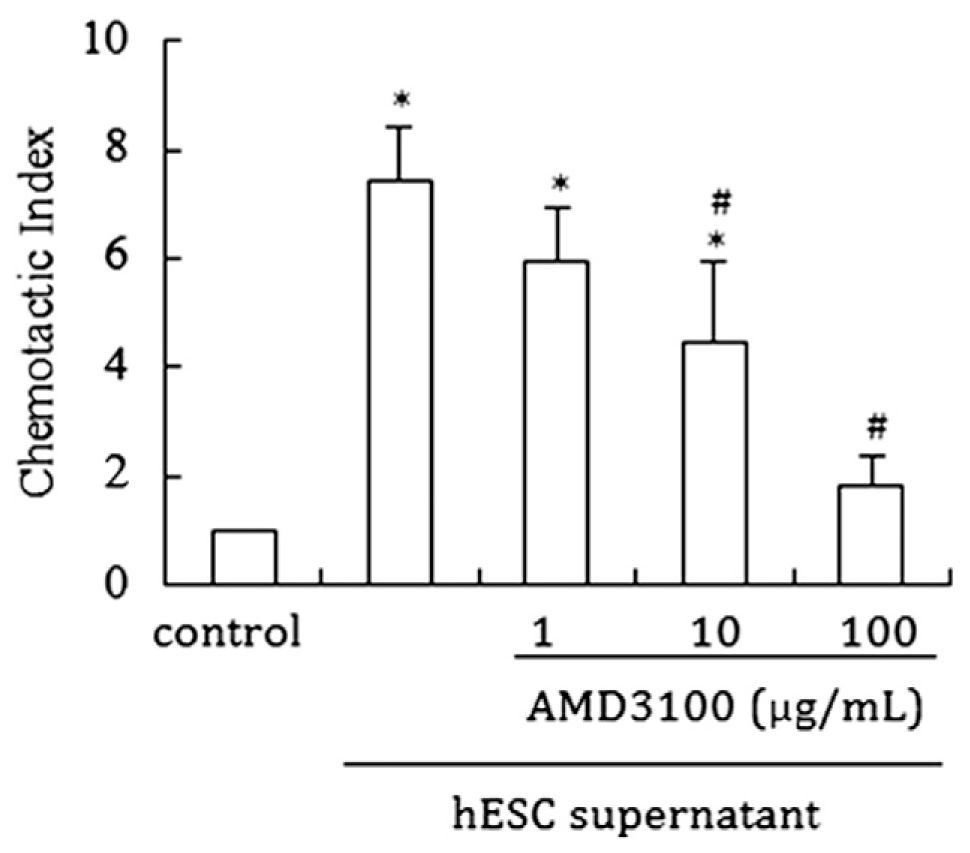
The chemotactic activity of conditioned supernatants to mBMC and hESCs. The migratory capacity of mBMC towards hESC supernatant. Untreated or AMD100 treated for 30 min mBMCs were seeded (5 × 10^4^/well) on the top of inserts and placed in a 24-well plate containing serum-free medium alone (control) or 48 h hESC serum-free supernatant. Data are shown as CI: cells migrating in response to the conditioned supernatants divided by cells responding to the serum-free medium. Bars represent the mean ± S.D. of four independent experiments, each performed in triplicate. * denotes a statistically significant difference (p b 0.05) vs control; # (p b 0.05) vs the cells without AMD3100 treatment.

A migration assay was carried out to determine the chemotactic activity of CXCL12, using the conditioned media. We detected the migratory capacity of mBMCs towards hESC supernatant and hESCs towards mBMC supernatant. As shown in Fig. 4, hESC supernatant significantly induced the mBMC migration. Pretreatment of mBMCs with the CXCR4 antagonist AMD3100 blocked the mBMC migration in a dose-dependent manner, and 100 μg/ml AMD3100 completely abolished the mBMC migration.

### Effects of E2 and P4 on the expression of CXCR4 and CXCL12 in mBMC and hESCs

**Figure 5:**
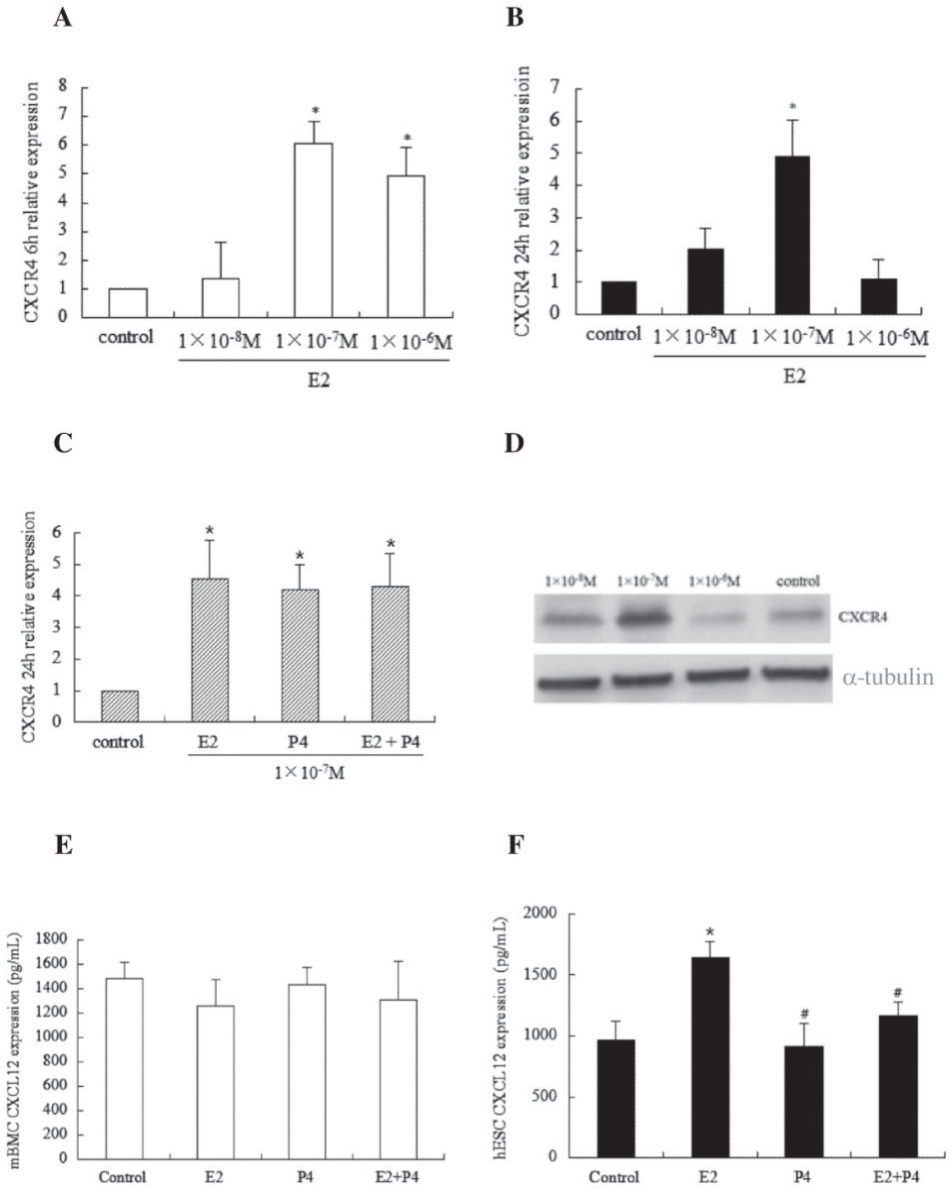
E2 and progesterone (P4) induced CXCR4 and CXCL12 expression in mBMC and hESCs. mBMCs were serum starved overnight prior to being stimulated with E2 or P4 in fresh serum-free DMEM/F12 medium for 6 h and 24 h and CXCR4 mRNA was analyzed by qRT-PCR. (A & B) E2-induced CXCR4 mRNA expression at 6 h and 24 h. (C) E2 alone or plus with P4-induced CXCR4 mRNA expression at 24 h. (D) Western blot analysis of E2-induced CXCR4 protein at 24 h. (E & F) mBMC and hESCs were serum starved overnight and supernatants were collected for ELISA analysis after the cells were stimulated with E2 or P4 for 48 h in serum-free DMEM/F12. The bars in each graph represent the mean ± S.D. of three individual experiments, each performed in triplicate. Statistical significance (*p b 0.05) is noted on the graphs (*p b 0.05 vs control, *p b 0.05 vs E2).

qPCR analysis demonstrated that E2 caused a significant increase in mRNA expression levels of CXCR4 in mBMCs in a dose dependent manner at 6 h but at 24 h only physiological levels of E2 (10^−7^ M) continued to drive CXCR4 expression in mBMCs (Figs. 5 A & B). As shown in Fig. 5C, progesterone (P4) alone at a physiological concentration of 10^−7^ M also induced CXCR4 in mBMCs. The combination of E2 and P4 resulted in a similar level of expression as treatment with either sex steroid alone. Ethanol was used as a vehicle to determine the non-specific effects on the CXCR4 expression. Ethanol treated cells does not showed any change in the CXCR4 expression comparing to control cells which are not treated either by ethanol or E2 or P4. Western blot analysis confirmed that CXCR4 protein induction was significantly increased (2.68-fold) after treatment with 10^−7^ M E2 for 24 h (Fig. 5D) while E2 at a concentration of 10^−8^ M showed no induction. Conversely, E2 at a concentration of 10^−6^ M did not increase CXCR4 protein levels compared to untreated cells. In summary, physiological concentrations of E2 and P4 results in increased expression of CXCR4 in mBMCs.

Based on the results of steroid-induced CXCR4 expression, physiological levels (10^−7^ M) of E2 and P4 were selected for examination of the effects of sex steroids on CXCL12 production. As shown in Fig. 5E, neither E2 nor P4 had any effect on CXCL12 production in mBMCs. However, in hESCs, E2 caused a significant increase in CXCL12 production compared to control and surprisingly P4 effectively inhibited E2-induced CXCL12 production in hESCs (Fig. 5F). In the both cell types mBMC and hESCs the protein levels were correlated to the mRNA levels confirmed by qRT-PCR.

### mBMC migration towards E2 and P4 treated hESC supernatants

**Figure 6:**
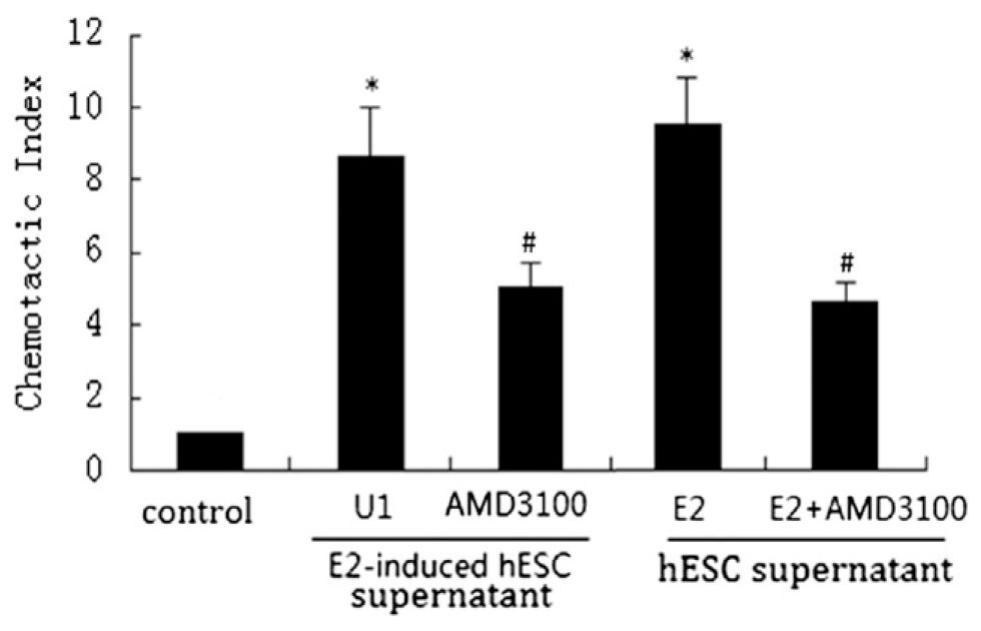
Effects of E2 on migratory capacity of mBMC. Untreated or E2-treated mBMCs were seeded (5 × 10^4^/well) on the top of inserts and placed in a 24-well plate containing serum-free DMEM/F12 or 48 h hESCs serum-free supernatant. The cells were pretreated with AMD3100 for 30 min before migration. Data are shown as CI: cells migrating in response to the conditioned supernatants divided by cells responding to the serum-free medium. Each bar represents the mean ± S.D. for data from three individual experiments and each experiment was performed in triplicate. * denotes statistical significance (p b 0.05) compared to control, and # denotes statistical significance (p b 0.05) compared to U1 or E2.

To determine if the enhanced CXCL12 and CXCR4 production induced by E2 would increase migration of BMCs to hESCs, we treated hESCs and mBMCs with E2 and performed a migration assay using the conditioned media, with and without the CXCR4 antagonist. The hESC supernatants were collected from 48 h old cultures. The mBMC in the upper insert were pretreated with 1 × 10^−7^ M E2 for 24 h after overnight serum starvation. Migration of mBMC was observed after 16 h. As shown in Fig. 6, mBMC migrated towards the E2-induced hESC supernatant in greater numbers compared to the untreated hESC supernatant. The number of migrated mBMCs decreased 42–51% when mBMCs were pretreated with the CXC4 antagonist AMD3100. The E2 induced migration of BMCs to hESCs was mediated by CXCL12/CXCR4.

## Discussion

Bone marrow derived stem cells migrate to the uterine endometrium of both mice and humans (Du and Taylor, 2007; Taylor, 2004). This migration likely has a key role in the repair of the uterus after damage. Indeed, our group has previously demonstrated that migration and engraftment of BM derived stem cells to the uterine endometrium is increased after ischemic injury and decreased by environmental toxins such as tobacco (Zhou et al., 2011; Wolff et al., 2011). Further, BMC delivery to mice after injury improved reproductive performance (Alawadhi et al., 2014) and mBMCs express several nuclear receptors (Wang et al., 2006; Zhou et al., 2001; Jeong and Mangelsdorf, 2009). Characterization of the chemokines that regulate stem cell engraftment may allow increased engraftment of endogenous stem cells injury. It has been previously shown in other tissues that increased CXCL12 production at a site of injury enhances stem cell recruitment and promotes functional recovery (Liu et al., 2013b; Penn et al., 2012; Sundararaman et al., 2011; Unzek et al., 2007). Here we demonstrate that bone marrow cells will migrate towards endometrial cell conditioned media; this chemoattraction of CXCR4 expressing bone marrow cells is similarly mediated by CXCL12 production by endometrial cells.

CXCL12 has been previously identified as an estrogen-regulated gene in estrogen receptor (ER)-positive ovarian and breast cancer cells (Jaerve et al., 2012). Here we show that in the endometrium, E2 significantly increased CXCL12 expression, suggesting a mechanism by which stem cells are recruited to the uterus in reproductive age women; it is likely that this recruitment ceases after menopause when the uterus is not longer needed for reproduction. Similarly, an increase in CXCR4 in bone marrow in response to estrogen enhances the mobility of these cells when needed for reproduction and in response to uterine signaling. Interestingly BM cells also produce CXCL12 at a high level. It is likely that local CXCL12 serves to retain these cells in the BM, preventing depletion. Elevated CXCL12 from the endometrium likely competes with BM derived CXCL12 as a chemoattractant for the BM stem cells (Segers et al., 2007). Elevated E2, which reaches the levels used here in the late proliferative phase, may ensure an adequate mobilization of stem cells near the time of ovulation and embryo implantation. P4 also induces the production of CXCL12 and may lead to further mobilization of stem cells in support of pregnancy.

The regulation of stem cells by sex steroids is likely a widespread phenomenon. Nakada et al. (Nakada et al., 2014) showed that E2 promotes the HSCs self-renewal and the replicative activity of the HSC pool is augmented in female versus male mice. Li et al. (2013) reported that E2 enhanced the recruitment of BM-derived EPC into infarcted myocardium and induced CXCR4 expression in mice. Similarly, Foresta et al. (2010) have observed an increase in the number of CXCR4^+^ EPC during the ovulatory phase, which was likely caused by E2 activation. Sex steroid induced alterations in stem cell renewal and mobilization may underlie many sex specific differences in health and disease.

In the ectopic endometrium of endometriosis, high E2 biosynthesis and low E2 inactivation lead to an excess of local E2 (Giudice and Kao, 2004). These provide a favorable condition for inducing BM-derived stem cell migration to normal and ectopic endometrium. Consistent with this theory, we have previously shown that stem cells are attracted to the ectopic endometrium (Sakr et al., 2014). The ectopic lesions compete for a limited pool of circulating BM-derived cells, depriving the uterus of the normal number of recruited stem cells. Insufficient uterine repair and regeneration may contribute to the infertility associated with endometriosis. The identification of CXCL12 as the primary chemokine that recruits BM-derived cells to the uterus may allow therapeutic use in endometriosis and other uterine disease to restore fertility. The expression of CXCL12 in mouse endometrial cells is far less than endometrial cells in humans. This may be the cause for the decrease in the number of stem cells recruited to the uterus in mouse. Moreover, mice do not menstruate and thereby may not be a need to attract new cells with every cycle while humans menstruate and have a greater need to regenerate the endometrium from stem cells. We conclude that estradiol plays a key role in normal and ectopic endometrium by augmenting the migration of BM-derived stem cells to the endometrium. Estradiol regulates stem cell migration by inducing CXCL12 expression by endometrial stromal cells and CXCR4 expression by BM-derived cells. Sex steroid induced stem cell recruitment may explain many health related sex differences. Estradiol or CXCL12/CXCR4 may prove to be useful therapeutic agents in stem cell mediated diseases.

## Abbreviations

hESCs: human endometrial stromal cells
BMCs: bone marrow cells
CXCR4: chemokine receptor type 4
E2: estradiol
CXCL12 or SDF-1α: stromal derived factor
BM: bone marrow
HSCs: hematopoietic stem cells
ESCs: endometrial stromal cells
EPCs: endothelial progenitor cells
MSCs: mesenchymal stromal cells
mBMCs: mouse bone marrow cells
ER: estrogen receptor
